# Supplementary material for: The structures of two archaeal type IV pili illuminate evolutionary relationships
Source: Nat Commun. 2020 Jul 9;11:3424. doi: 10.1038/s41467-020-17268-4 (PMC7347861; doi:10.1038/s41467-020-17268-4)
Supplement: Supplementary file 1 — Supplementary Information [file 41467_2020_17268_MOESM1_ESM.pdf]

Supplementary Information

The Structures of Two Archaeal Type IV Pili Illuminate Evolutionary Relationships

Fengbin Wang, Diana P. Baquero et al.

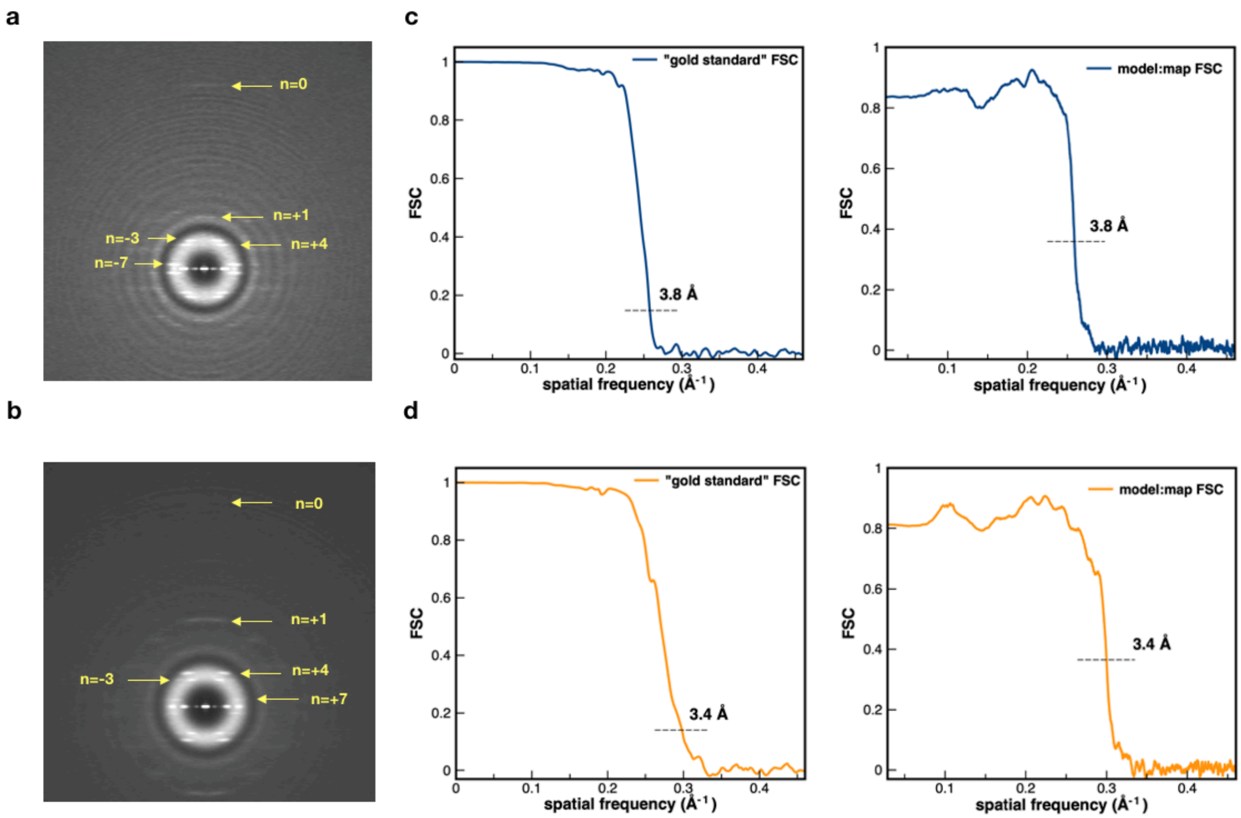

Supplementary Fig. 1 | Power spectrum and Fourier Shell Correlation (FSC) calculations
a-b, The averaged power spectrum from *P. arsenaticum* pili is shown in (**a**) and from the *S. solfataricus* pili in (**b**). The averages were computed from ~1,000 non-overlapping segments, each 512 px long. The layer lines that were used to estimate the helical symmetry are labelled. **c-d**, Resolution estimates for *P. arsenaticum* (**c**) and *S. solfataricus* pili (**d**). On the left are the map:map “gold standard” FSCs using the 0.143 criterion. On the right are the model:map FSC calculations using a 0.38 criterion, which is sqrt (0.143).

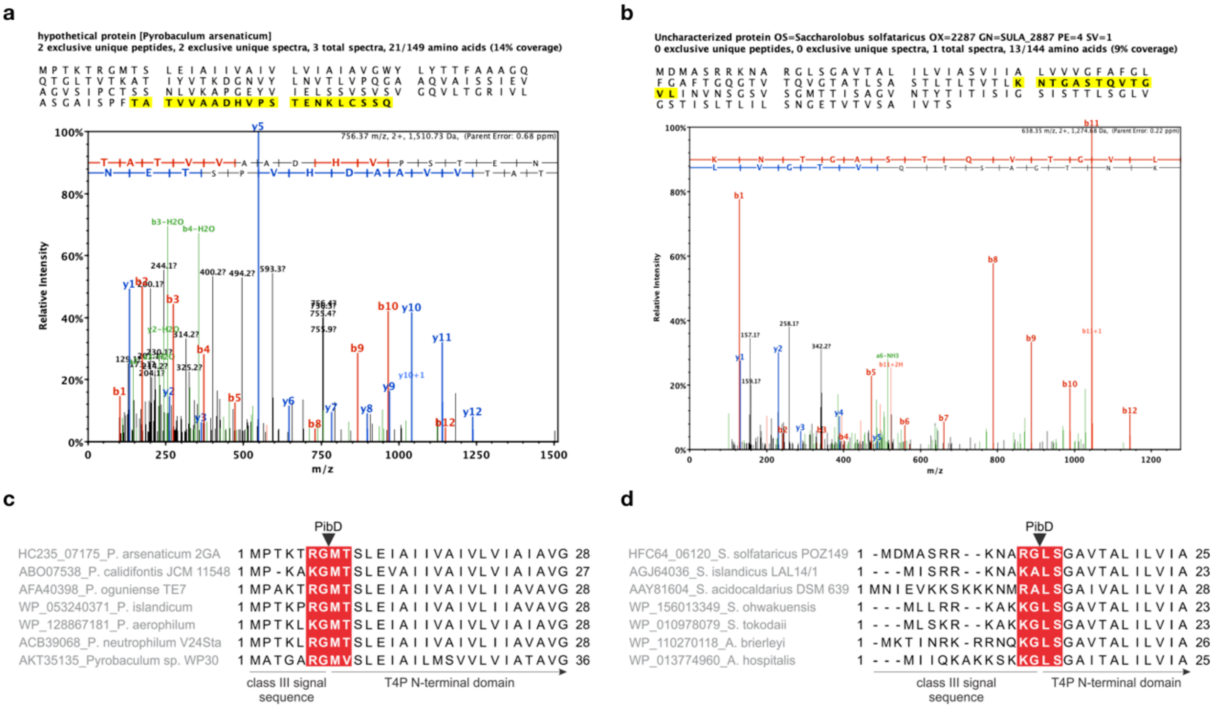

Supplementary Fig. 2 | Mass spec verification

a-b, MS/MS analysis detected a unique peptide of the pilin molecule that was fit into the cryo-EM map (the *P. arsenaticum* pilin in **a** and the *S. solfataricus* pilin in **b**).

c-d, The cleavage sites of the signal peptides in prepilins (*P. arsenaticum* in **c** and *S. solfataricus* in **d**).

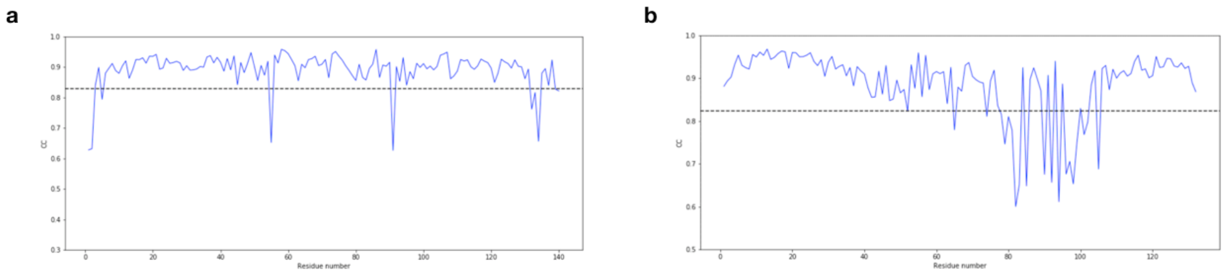

Supplementary Fig. 3 | Real-space correlations of the model

a-**b**, Per-residue real-space correlation coefficient (RSCC) plot of the atomic model (the *P. arsenaticum* pilus in **a** and the *S. solfataricus* pilus in **b**) against the corresponding non-masked cryo-EM map. The drops of the CC for some residues are due to glycosylation.

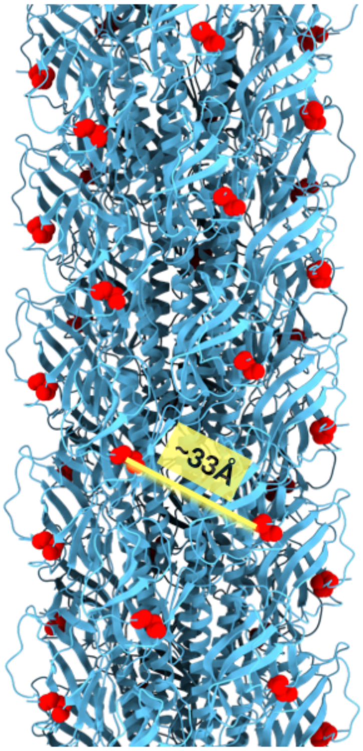

Supplementary Fig. 4 | cysteine residues on the *P. arsenaticum* pilus

The *P. arsenaticum* pilus ribbon model is shown with cysteine residues as red spheres.

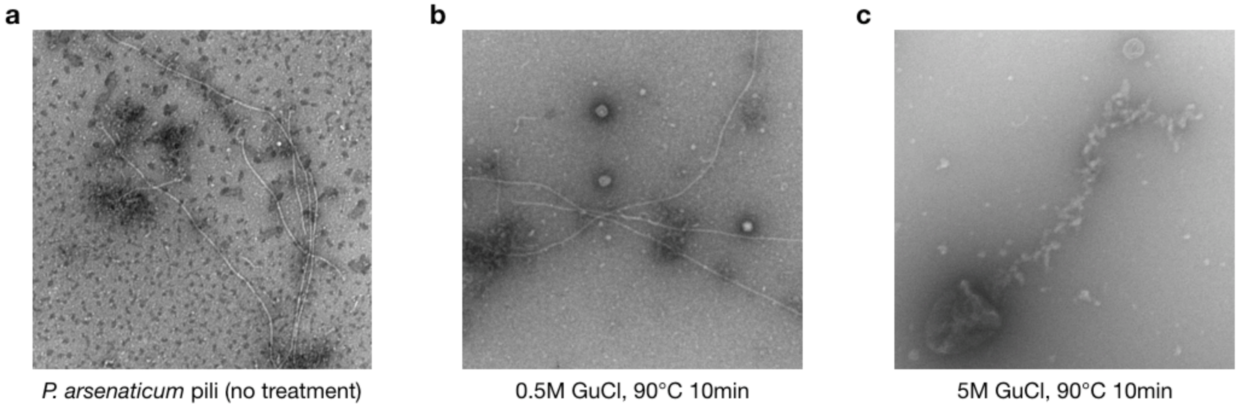

Supplementary Fig. 5 | Stability of the *P. arsenaticum* pilus

a, Negative staining of the *P. arsenaticum* pilus in a buffer comprises 20 mM KH_2PO_4, 100 mM NaCl, 2 mM $MgCl_2$, 0.5 mM $Ca(NO_3)_2$.

b, Negative staining of the *P. arsenaticum* pilus after boiling in 0.5M guanidinium-HCl for 10min.

c, Negative staining of the *P. arsenaticum* pilus after boiling in 5M guanidinium-HCl for 10min.

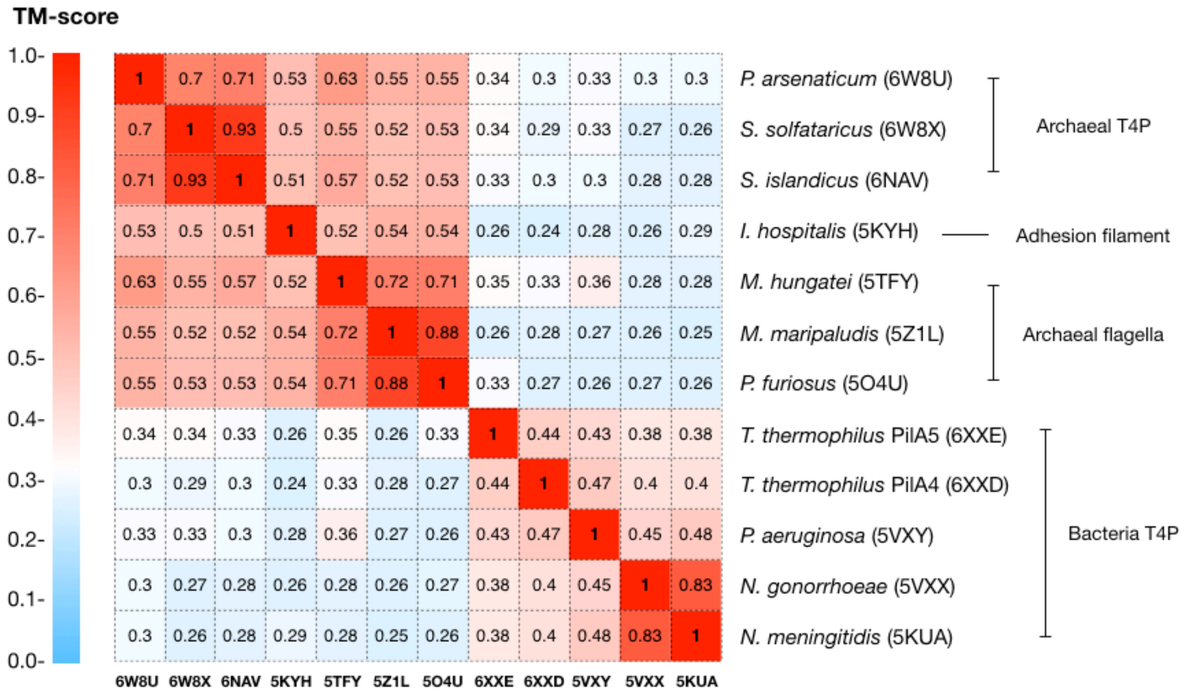

Supplementary Fig. 6 | TM-score matrix of globular domains

All-against-all comparison of the bacterial T4P and archaeal structures with N-terminal T4P domains. The N-terminal helices have been removed and only the globular domains were analyzed. The matrix is based on the pairwise TM-score from TM-align server. TM-score is a metric for measuring the similarity of two protein structures, and it has the value in the range from 0 to 1, where 1 indicates a perfect match between two structures. When 0.0 < TM-score < 0.30, it suggests random structural similarity; while 0.5 < TM-score < 1.00, suggests two proteins have approximately the same fold.

Supplementary Table 1 | Comparison between *S. islandicus, S. solfataricus*, and *P. arsenaticum* pilins

Pilin	*S. islandicus*	*S. solfataricus*	*P. arsenaticum*
Pilin length (excluding signal peptide)	131	132	142
Charged residues (%)	1.5%	2.4%	9.1%
O-linked glycosylation sites (observed by EM)	6	7	0
N-linked glycosylation sites (Observed by EM)	0	0	1
Sequence identity (To *S. islandicus* pilin)	100%	81%	18%

Supplementary Table 2 | List of initial helical symmetries tested (*P. arsenaticum pili*)

List of possible symmetries	Helical rise (Å)	Helical twist (degrees)	Point group
1	5.3	101.7	C1
2	7.3	141.8	C1
3	12.1	-126.1	C1
4	12.1	-63.0	C2
5	9.0	-93.4	C1
6	7.1	-74.1	C1
